# Bisulfite-free and base-resolution analysis of 5-methylcytidine and 5-hydroxymethylcytidine in RNA with peroxotungstate†
†Electronic supplementary information (ESI) available. See DOI: 10.1039/c9cc00274j


**DOI:** 10.1039/c9cc00274j

**Published:** 2019-01-30

**Authors:** Fang Yuan, Ying Bi, Paulina Siejka-Zielinska, Ying-Lin Zhou, Xin-Xiang Zhang, Chun-Xiao Song

**Affiliations:** a Ludwig Institute for Cancer Research and Target Discovery Institute , Nuffield Department of Medicine , University of Oxford , OX3 7FZ , UK . Email: chunxiao.song@ludwig.ox.ac.uk; b Beijing National Laboratory for Molecular Sciences (BNLMS) , MOE Key Laboratory of Bioorganic Chemistry and Molecular Engineering , College of Chemistry , Peking University , Beijing 100871 , China . Email: zxx@pku.edu.cn

## Abstract

5-Methylcytosine (5mC) and 5-hydroxymethylcytosine (5hmC), two of the best-studied DNA modifications, play crucial roles in normal development and disease in mammals. Although 5-methylcytidine (m^5^C) and 5-hydroxymethylcytidine (hm^5^C) have also been identified in RNA, their distribution and biological function in RNA remain largely unexplored, due to the lack of suitable sequencing methods. Here, we report a base-resolution sequencing method for hm^5^C in RNA. We applied the selective oxidation of hm^5^C to trihydroxylated-thymine (^th^T) mediated by peroxotungstate. ^th^T was subsequently converted to T during cDNA synthesis using a thermostable group II intron reverse transcriptase (TGIRT). Base-resolution analysis of the hm^5^C sites in RNA was performed using Sanger sequencing. Furthermore, in combination with the TET enzyme oxidation of m^5^C to hm^5^C in RNA, we expand the use of peroxotungstate oxidation to detect m^5^C in RNA at base-resolution. By using this method, we confirmed three known m^5^C sites in human tRNA, demonstrating the applicability of our method in analyzing real RNA samples.

## Abstract 2

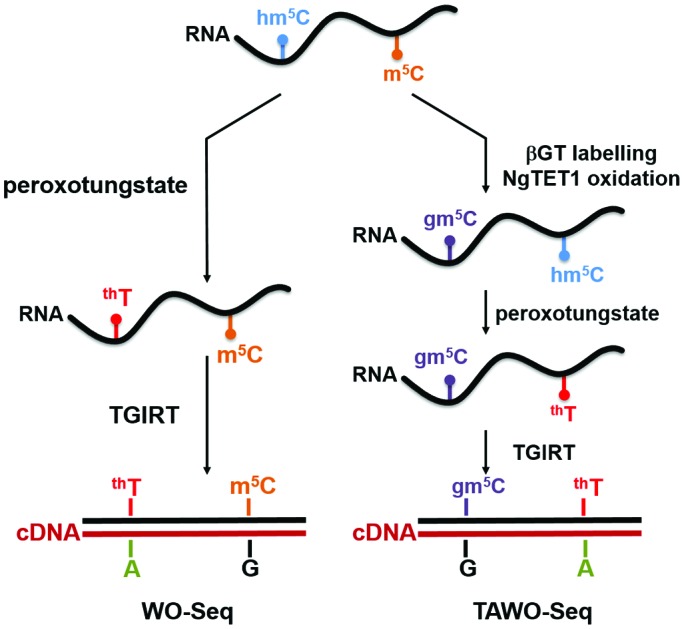
WO-Seq: a bisulfite-free and base-resolution sequencing method based on peroxotungstate oxidation is presented for the identification of hm^5^C sites in the transcriptome. Combining the peroxotungstate oxidation with TET enzyme oxidation, m^5^C can also be detected in a procedure termed TET-Assisted WO-Seq (TAWO-Seq).

## 

Epitranscriptome, which refers to the multitude of RNA chemical modifications, has vital roles in post-transcriptional gene regulation.^1^–^3^ 5-Methylcytidine (m^5^C) and 5-hydroxymethylcytidine (hm^5^C) are two of the major RNA modifications in eukaryotic cells, however, our understanding of them is still in its infancy. M^5^C is abundant in noncoding RNA, and has the ability to stabilize tRNA secondary structure,^4^–^6^ but the knowledge about its distribution and function in mRNA are still very limited due to the inconsistent results obtained from the current sequencing methods.^7^–^9^ Hm^5^C has been shown to be enriched in *Drosophila melanogaster* mRNA, increase mRNA translation and play a central role in Drosophila brain development.^10^ Hm^5^C also exists in mammalian RNA, albeit at low levels,^11^ and the TET proteins that oxidize 5-methylcytosine (5mC) to 5-hydroxymethylcytosine (5hmC) in DNA can also do so in RNA.^10^,^12^ However, the distribution and regulation roles of hm^5^C in the mammalian transcriptome remain unknown due to the lack of sensitive and robust sequencing methods.

The most common way to sequence m^5^C in RNA is to adopt bisulfite sequencing, which is widely used to sequence 5mC in DNA. Bisulfite treatment deaminates unmethylated cytosine to uracil in single-strand RNA, while leaving m^5^C unconverted. Therefore, bisulfite sequencing provides base-resolution information of m^5^C. Using bisulfite sequencing, widespread m^5^C sites were identified in both coding and non-coding RNAs.^7^,^8^ However, bisulfite treatment employs sequential thermal acidic and alkaline conditions that severely damage the RNA. Further analysis also revealed potential false positives from RNA bisulfite sequencing due to incomplete conversion of unmethylated cytosine in the double-stranded RNA regions and other modifications resistant to bisulfite treatment.^13^–^15^ Other methods to sequence m^5^C in RNA are immunoprecipitation-based that use m^5^C-specific antibodies or methyltransferases to pull down m^5^C-containing RNA.^16^–^18^ These methods, however, do not have base-resolution and lose the quantitative levels of m^5^C. Mapping hm^5^C in RNA is even more challenging. To date, there is no base-resolution sequencing method for hm^5^C. The only reported method is the antibody-based immunoprecipitation approach.^10^ This method has been applied to the Drosophila transcriptome, but has yet to be successful in the mammalian transcriptome. Clearly, new RNA-friendly and high-resolution sequencing methods are highly desirable to further study the elusive distribution, localization and biological roles of these two modifications in RNA. Here, we report bisulfite-free and base-resolution sequencing methods for hm^5^C and m^5^C based on peroxotungstate oxidation.

Peroxotungstate oxidation was first developed by the Okamoto group^19^,^20^ for selective oxidation of 5hmC in DNA to trihydroxylated-thymine (^th^T). ^th^T is a thymine derivative, and will induce C-to-T transition in DNA after PCR. However, the peroxotungstate oxidation reaction requires single-strand DNA. The reaction is strongly inhibited in double-strand DNA with a conversion rate of less than 10%, which severely limits its application.^20^ Although this reaction is not suitable for DNA samples, we hypothesized that it could be ideal to detect hm^5^C in RNA, which is mostly single-stand (Fig. 1a). We termed this approach peroxotungstate oxidation sequencing (WO-Seq).

**Fig. 1:**
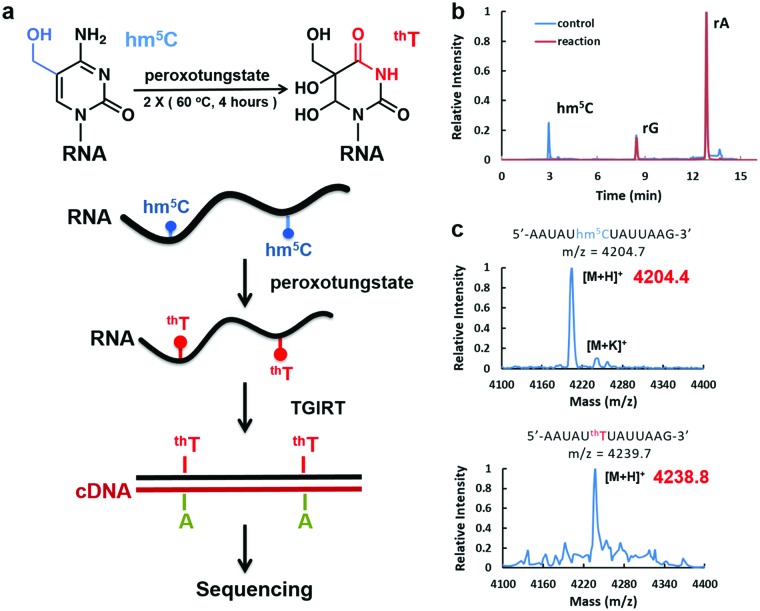
Peroxotungstate reaction on hm^5^C-containing RNA. (a) Illustration of the peroxotungstate reaction and workflow of WO-Seq. Hm^5^C-containing RNA is specifically oxidized by peroxotungstate, and then reverse transcribed by thermostable group II intron reverse transcriptase (TGIRT). The oxidation product of hm^5^C (^th^T) is converted to T during cDNA synthesis, thus can be used for base-resolution sequencing of hm^5^C in RNA. (b) HPLC-MS/MS results of the hydrolysed product of synthesized hm^5^C-containing RNA1 before and after the peroxotungstate reaction. Peaks of adenosine (rA), guanosine (rG) and hm^5^C are labelled in the figure. (c) MALDI-MS characterization of an hm^5^C-containing RNA fragment of RNA1 treated with peroxotungstate. Calculated *m*/*z* is shown in black, observed *m*/*z* is shown in red.

We started with optimizing the oxidation conditions of the peroxotungstate against in vitro-transcribed hm^5^C-containing RNA1. MALDI-TOF MS and HPLC-MS/MS were used to monitor the reaction rate. After two rounds of 4 hours incubation at 60 °C, the hm^5^C peak in HPLC-MS/MS was undetectable (Fig. 1b), and the MALDI peak of RNA fragments containing one hm^5^C changed from *m*/*z* = 4204.4 to *m*/*z* = 4238.8. This is consistent with the calculated *m*/*z* change from hm^5^C-containing RNA to ^th^T-containing RNA (Fig. 1c). Sensitivity of the peroxotungstate treatment for hm^5^C was also tested (Fig. S1, ESI†). Samples of different combination of hm^5^C modified RNA and unmodified RNA were treated by peroxotungstate, and then analysed by HPLC-MS/MS. The conversion rates of hm^5^C were similar in all samples, indicating that the peroxotungstate treatment is suitable for real biological samples which has low hm^5^C content.

Next, we investigated the potential of the hm^5^C-to-T transition during cDNA synthesis using the peroxotungstate-oxidized RNA template. We designed and synthesized a 73mer RNA that contained three hm^5^C sites (RNA2). To enable us to monitor the efficiency of the hm^5^C-to-T conversion, one hm^5^C was positioned so that, upon successful hm^5^C-to-T conversion, a Taq^α^I restriction enzyme recognition site in the resulting RT-PCR product was destroyed (Fig. 2a). Since ^th^T is not a natural occurring base, we first sought to investigate its behavior during cDNA synthesis. Several commercially available reverse transcriptases were tested on this RNA template. Interestingly, only the thermostable group II intron reverse transcriptase (TGIRT)^21^,^22^ could read though all reacted hm^5^C sites, while Superscript III and Bst 3.0 DNA polymerase induced truncations at the reacted hm^5^C sites (Fig. 2b). The reaction conditions of TGIRT were further optimized to get the best reverse transcription efficiency, and after subsequent PCR, the DNA products were digested with Taq^α^I. As shown in Fig. 2c, 67% of the PCR products from the oxidized hm^5^C-containing RNA2 sample stayed intact after the Taq^α^I treatment, indicating loss of restriction enzyme cut site and the successful base change induced by the peroxotungstate-oxidized RNA during cDNA synthesis.

**Fig. 2:**
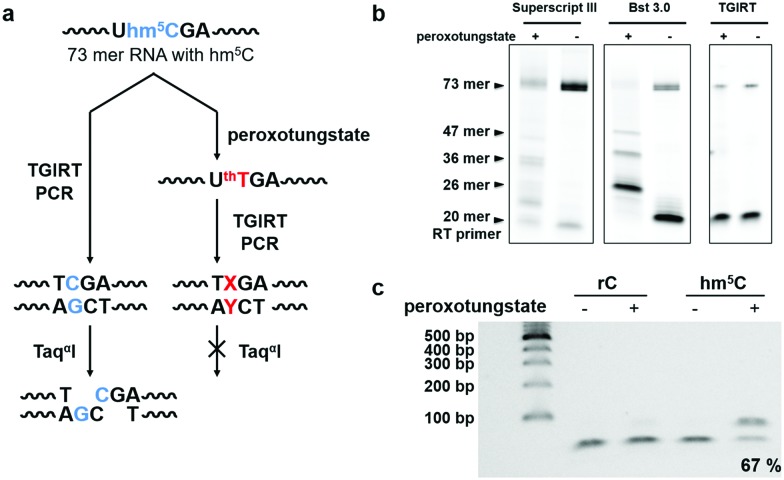
Restriction enzyme digestion assay showed effective base change during cDNA synthesis using the peroxotungstate-oxidized RNA template. (a) Illustration of the restriction enzyme digestion assay for the investigation of the base change mediated by peroxotungstate. X represent T or A or G, while Y is the complementary base of it. (b) Reverse transcription products of hm^5^C-containing RNA2 before and after peroxotungstate treatment using different reverse transcriptases. Hm^5^C-containing RNA2 has three hm^5^C sites at position 26, 36 and 47. The full length is 73 mer. (c) RT-PCR product of the 73-mer model RNA2 containing a Taq^α^I cut site. Samples without peroxotungstate treatment and control normal cytidine (rC) containing RNA2 treated with peroxotungstate were cleaved completely. About 67% of the reacted hm^5^C-RNA amplified product stayed intact, indicating the loss of the restriction enzyme cut site and the successful base change.

We then performed Sanger sequencing of the PCR product from the oxidized RNA samples (Fig. 3). At each hm^5^C site, a new peak of thymine signal appears, confirming the base change is indeed C-to-T. In order to accurately quantify the C-to-T conversion rate, the PCR product was cloned and sequenced individually. A 62.1% conversion rate was observed from a total of 66 hm^5^C sites sequenced (Fig. S2, ESI†), consistent with the restriction enzyme digestion result. As a control, PCR products of peroxotungstate-treated normal cytosine (rC)-containing RNA2 and m^5^C-containing RNA2 were also cloned and sequenced (Fig. S3, ESI†). Results showed that both rC and m^5^C sites did not change after the treatment, indicating an excellent selectivity of peroxotungstate oxidation on hm^5^C. Notably, peroxotungstate oxidation is a mild reaction, which showed less damage on RNA compared with bisulfite reaction (Fig. S4, ESI†).

**Fig. 3:**
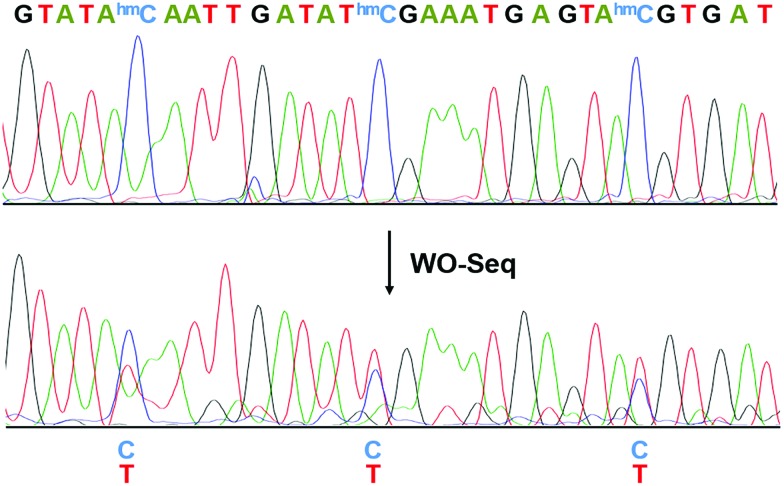
Electropherograms of Sanger sequencing results before and after WO-Seq. The conversion of C-to-T happened at each hm^5^C site.

After demonstrating WO-Seq for hm^5^C sequencing, we next sought to expand its use for m^5^C sequencing in RNA. In DNA, 5hmC is generated by the oxidation of 5mC mediated by the TET enzyme. Recently, the mammalian TET enzyme was reported to have the ability of oxidizing m^5^C to hm^5^C in RNA.^12^ We tested Naeglaria Tet-like oxygenase (NgTET1)^23^ and showed it can also oxidize m^5^C to hm^5^C on m^5^C-containing RNA1 by both MALDI-MS and HPLC-MS/MS (Fig. S5, ESI†). Based on this, we further aimed to combine the peroxotungstate oxidation with NgTET1 oxidation to detect m^5^C in a procedure we termed TET-Assisted WO-Seq (TAWO-Seq) (Fig. 4a). The results of both oxidation reactions were verified by HPLC-MS/MS (Fig. S6, ESI†). Restriction enzyme digestion assays and Sanger sequencing were performed (Fig. 4b and c). Sanger sequencing results showed the C-to-T transition at each m^5^C site. The m^5^C-to-T conversion rate was 50% estimated by restriction enzyme analysis, lower than that of hm^5^C, due to incomplete m^5^C to hm^5^C oxidation by NgTET1 (Fig. S6, ESI†). We also cloned and sequenced individual PCR product for the m^5^C sample. As shown in Fig. S7 (ESI†), 33.3% of the total m^5^C sites were successfully detected. Commercially available mouse Tet1 (mTet1) was also tested for the TAWO-Seq, which gives similar results with NgTET1 (Fig. S8a, ESI†). We further demonstrated that β-glucosyltransferase (βGT) can label hm^5^C with glucose and thereby protect it from peroxotungstate oxidation (Fig. S9, ESI†). Combining βGT protection with TAWO-Seq could therefore enable it to detect m^5^C specifically.

**Fig. 4:**
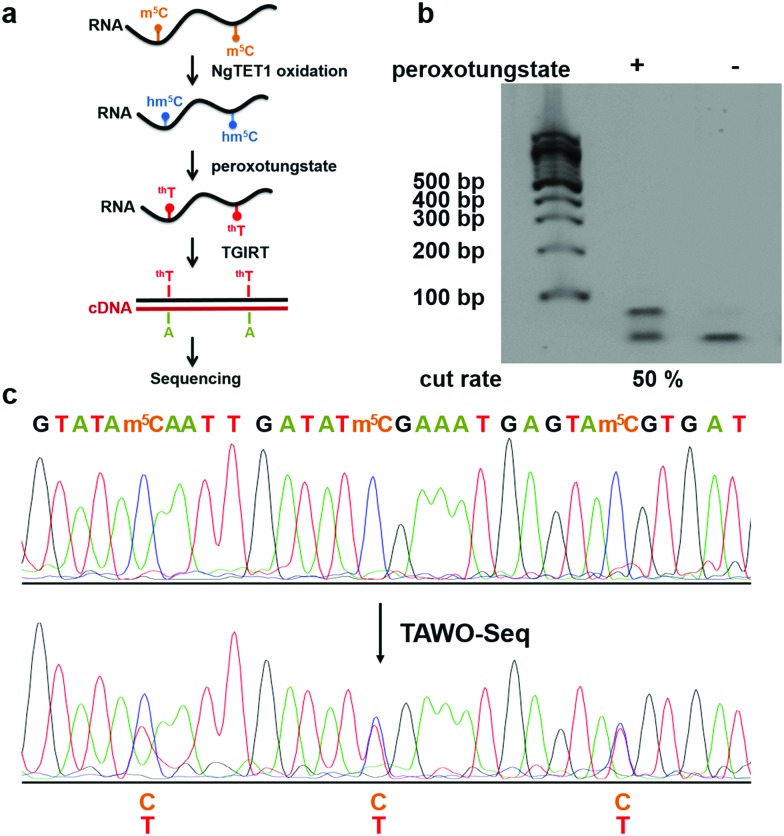
The combination of NgTET1 oxidation and peroxotungstate reaction in detecting m^5^C in RNA in TAWO-Seq. (a) Illustration of TAWO-Seq strategy for the identification of m^5^C in RNA at single-nucleotide resolution. (b) Restriction enzyme digestion assay of (+) and (–) NgTET1-assisted peroxotungstate-treated samples. About 50% of the m^5^C sites were detected. (c) Sanger-sequencing results before and after TAWO-Seq.

To further demonstrate the utility of TAWO-Seq on real RNA sample, we applied it to the endogenous tRNA^Asp(GUC)^ in 293T cells. The tRNA^Asp(GUC)^ contains three known m^5^C sites at structural positions 38, 47 and 48 (Fig. S10a, ESI†).^24^–^26^ Both NgTET1 and mTet1 were used to oxidize the tRNA and the products were then treated with peroxotungstate. The RT-PCR product of treated tRNA^Asp(GUC)^ was cloned and sequenced. As shown in Fig. S10b and c (ESI†), 35.2% of the m^5^C sites were successfully detected with NgTET1 assisted WO-Seq, and 37.5% of the m^5^C sites were detected using mTet1 assisted WO-Seq, which demonstrated the applicability of TAWO-Seq to real RNA samples. Among the three m^5^C sites in tRNA^Asp(GUC)^, we found that m^5^C at position 48 has the highest C-to-T conversion rate (68.4% by NgTET1 assisted WO-Seq, 77.8% by mTet1 assisted WO-Seq). According to the tRNA^Asp(GUC)^ structure, this site is in a double-stranded CpG context, which is an ideal substrate of TET enzyme.^23^ It is likely that the different m^5^C-to-T conversion rates of three m^5^C sites are caused by the sequence preference of the TET proteins.

In conclusion, we have described WO-Seq as an RNA friendly, chemical oxidation-based, base-resolution method to sequence hm^5^C in RNA. We demonstrate the specific hm^5^C-to-T transition using peroxotungstate to oxidize the RNA followed by cDNA synthesis with the TGIRT enzyme, and Sanger sequencing results have proved the base-resolution sequencing ability of this method. We further demonstrate the ability of TAWO-Seq to detect m^5^C by combining WO-Seq with the prior NgTET1 or mTet1 oxidation of m^5^C to hm^5^C. The successful detection of m^5^C sites in human tRNA demonstrates our method is applicable for real RNA samples. Both WO-Seq and TAWO-Seq could potentially solve the false positive issue of bisulfite sequencing since they directly detect modified cytosine without affecting unmodified cytosine. Further improvement of both methods to increase the conversion rate and apply to mRNA samples using next-generation sequencing technology are underway in the lab. We believe this method could be highly useful for the identification of unexplored m^5^C/hm^5^C distribution and function in the transcriptome.

We would like to acknowledge P. Spingardi, G. Berridge and B. Kessler for helping with the HPLC-MS/MS; C. He for the mTet1; F. Howe for editing the manuscript. This work was supported by the Ludwig Institute for Cancer Research. Work in the C.-X. Song lab is also supported by Cancer Research UK (C63763/A26394 and C63763/A27122), NIHR Oxford Biomedical Research Centre, and Conrad N. Hilton Foundation. The views expressed are those of the authors and not necessarily those of the NHS, the NIHR or the Department of Health. F. Yuan and Y. Bi are supported by China Scholarship Council.

## Conflicts of interest

There are no conflicts to declare.

## Supplementary Material

Supplementary information
