# Supplementary material for: Bisulfite-free and base-resolution analysis of 5-methylcytidine and 5-hydroxymethylcytidine in RNA with peroxotungstate
Source: Chem Commun (Camb). 2019 Jan 30;55(16):2328–31. doi: 10.1039/c9cc00274j (PMC6984333; doi:10.1039/c9cc00274j)
Supplement: Supplementary file 1 [file CC-055-C9CC00274J-s001.pdf]

Electronic Supplementary Material (ESI) for ChemComm.
This journal is © The Royal Society of Chemistry 2019

Supporting Information

Bisulfite-free and base-resolution analysis of 5-methylcytidine and 5-hydroxymethylcytidine in RNA with peroxotungstate

Fang Yuan,[a,b] Ying Bi,[a] Paulina Siejka-Zielinska,[a] Ying-Lin Zhou,[b] Xin-Xiang Zhang,[b] and Chun-Xiao Song*[a]*

a. Ludwig Institute for Cancer Research and Target Discovery Institute, Nuffield Department of Medicine, University of Oxford. OX3 7FZ, UK

E-mail: chunxiao.song@ludwig.ox.ac.uk.

b. Beijing National Laboratory for Molecular Sciences (BNLMS), Key Laboratory of Bioorganic Chemistry and Molecular Engineering of Ministry of Education, College of Chemistry, Peking University, Beijing 100871, China

E-mail: zxx@pku.edu.cn

Table of Contents

Experimental Procedures **4**

Preparation of model RNA oligo 4

Model RNA oxidation with peroxotungstate 4

Sensitivity and RNA degradation test 4

MALDI-MS analysis and HPLC-MS analysis 4

Reverse transcription, PCR and restriction enzyme digestion assay 5

T-A cloning and Sanger sequencing 5

NgTET1 oxidation, mTET1 oxidation and peroxotungstate reaction of m^{5}C-RNA 6

βGT labelling of hm^{5}C in RNA 6

Cell culture and microRNA isolation 6

NgTET1 oxidation, mTET1 oxidation and peroxotungstate reaction of tRNA from 293T cells 7

Supplementary Tables **8**

Table S1. Oligonucleotides 8

Table S2. Compound-dependent LC-MS/MS parameters used for nucleosides quantification 9

Supplementary Figures **10**

Figure S1. Hm^{5}C conversion rate of different combinations of hm^{5}C modified and unmodified RNA samples. 10

Figure S2. Sanger sequencing results of PCR product from peroxotungstate treated hm^{5}C-containing RNA2 11

Figure S3. Sanger sequencing results of PCR products from peroxotungstate treated rC-containing RNA2 and m^{5}C-containing RNA2 12

Figure S4. Gel results of (1) control total RNA, (2) total RNA after bisulfite treatment and (3) total RNA after peroxotungstate treatment. 13

Figure S5. MALDI-MS and HPLC-MS/MS results of m^{5}C-containing RNA1 before and after NgTET1 oxidation 14

Figure S6. HPLC-MS/MS results of NgTET1-assisted peroxotungstate treated m^{5}C-containing RNA2 15

Figure S7. Sanger sequencing analysis of individual PCR product of m^{5}C-containing RNA2 after the NgTET1 assisted peroxotungstate treatment 16

Figure S8. The combination of mTET1 oxidation and peroxotungstate reaction in detecting m^5C in model RNA.. 17

Figure S9. Using β- glucosyltransferase (βGT) to label hm^5C with glucose can protect the hm^5C-containing RNA from peroxotungstate oxidation.... 18

Figure S10. TAWO-Seq results of $tRNA^{Asp(GUC)}$ from 293T cells.. 19

Experimental Procedures

Preparation of model RNA oligo

60-mer model RNA and 73-mer model RNA with C, m^5C or hm^5C were generated by the HiScribeTM T7 High Yield RNA Synthesis Kit using CTP, 5-Methoxycytidine-5'-Triphosphate or 5-Hydroxymethylcytidine-5'-Triphosphate (Trilink Biotech), along with ATP, GTP and UTP. Synthesized RNA was purified by phenol-chloroform extraction and ethanol precipitation. RNA was then dissolved in 50 μL DNase/RNase free water (Invitrogen) and the concentration and quality were checked using NanoDrop™ 2000/2000c Spectrophotometers (ThermoFisher) and NovexTM 15% TBE-Urea Gel (ThermoFisher). 1 μL RiboLock RNase Inhibitor (Thermo) was added to the purified model RNA, and all of the RNA was stored at – 80 ^oC.

Oxidation of model RNA by peroxotungstate

Dinuclear peroxotungstate ($K_2[\{W(=O)(O_2)_2(H_2O)\}_2(\mu\text{-}O)]\bullet 2H_2O$) was prepared according to the reported procedures (*Chem. Soc.* Dalton Trans., **1989**, 1203). The synthesized oxidant was stored at room temperature. Generally, 2 μg model hm^5C-containning RNA was incubated in a working solution of 5 mM peroxotungstate, 1 U / μL RNase inhibitor, 100 mM sodium chloride in 200 mM sodium phosphate buffer (pH = 7.0) at 60 ^oC. After 4 hours, model RNA was purified on Zymo-Spin columns (Zymo Research) and eluted in 10 μL DNase/RNase free water. Then, the reacted RNA was incubated in the same oxidation condition for another 4 hours, and purified by Micro Bio-Spin 6 column (Bio-Rad). Qubit^{TM} RNA HS Assay Kit (Thermo Fisher) was used to measure the concentration, and the product was stored at -20^oC waiting for further analysis.

Sensitivity and RNA degradation test

RNA samples of different combination of hm^5C-containing RNA1 and normal rC-containing RNA1 were prepared, which contained 0%, 25%, 50%, 75% and 100% of hm^5C-containing RNA1. These samples were treated by peroxotungstate oxidation described above, and then analyzed by HPLC-MS.

Total RNA of mESC was extracted using TRIzol^{TM} Reagent (Invitrogen^{TM}). Same amount of the total RNA sample was treated by peroxotungstate or EZ RNA Methylation^{TM} Kit (Zymo Research). Control untreated RNA and these treated RNA were analyzed by agarose gel.

MALDI-MS analysis and HPLC-MS analysis

Oxidized product of hm^5C-containing RNA1 was analyzed by MALDI-MS and HPLC-MS/MS. For MALDI-MS, about 200 ng oxidized RNA was digested using 1 μL 1000 U / μL RNase T1 (Thermo Fisher) at 37 ^oC for 20 min, and purified by desalting resins. Then, the digested RNA fragments were characterized by Voyager-DE MALDI-TOF (matrix-assisted laser desorption ionization time-of flight) Biospectrometry Wrokstation.

For HPLC-MS/MS, about 50 ng RNA sample was digested using DNA degradase plus^{TM} (1 U / μL,

Zymo Research) in the presence of 40 nM deaminase inhibitors erythro-9-amino-β-hexyl-α-methyl-9H-purine-9-ethanl hydrochloride (Sigma-Aldrich). After incubation at 37 ^{o}C for 2 hours, equal volume of solvent A (10 mM ammonium acetate, pH = 6.0) of the HPLC method was added to the solution, and the resulting solution was filtered with Amicon Ultra-0.5 mL 10 K centrifugal filters (Merck Millipore) to remove the proteins.

The HPLC-MS/MS analysis was carried out with 1290 Infinity LC Systems (Agilent) coupled with a 6495B Triple Quadrupole Mass Spectrometer (Agilent). A ZORBAX Eclipse Plus C18 column (2.1 x 150mm, 1.8-Micron, Agilent) was used. The column temperature was maintained at 40 ^{o}C, and the solvent system was water containing 10mM ammonium acetate (pH 6.0, solvent A) and methanol (solvent B) with 0.4 mL/min flow rate. The gradient was: 0-5 min; 0 % solvent B; 5-8 min; 0-5.63 % solvent B; 8-9 min; 5.63 % solvent B; 9-16 min; 5.63-13.66 % solvent B; 16-17 min; 13.66-100 % solvent B; 17-21 min; 100 % solvent B; 21-24.3 min; 100-0 % solvent B; 24.3-25 min; 0 % solvent B. The dynamic multiple reaction monitoring mode (dMRM) of the MS was used for quantification. The source-dependent parameters were as follows: gas temperature 230 ^{o}C, gas flow 14 L/min, nebulizer 40 psi, sheath gas temperature 400 ^{o}C, sheath gas flow 11 L/min, capillary voltage 1500 V in the positive ion mode, nozzle voltage 0 V, high pressure RF 110 V and low pressure RF 80 V, both in the positive ion mode. The fragmentor voltage was 380 V for all componds, while other compound-dependent parameters were summarized in **Table** S2.

Reverse transcription, PCR and restriction enzyme digestion assay.

100 ng RNA sample was used for each reverse transcription reaction. For TGIRT enzyme, the template RNA was mixed with 250 nM FAM-labelled primer, FAM-TTCCCTTACCTACCACTTCC, 10 mM DTT, 1 U / μL RNase inhibitor and 560 U TGIRT (InGex, St. Louis, MO) in 450 mM NaCl, 5 mM $MgCl_2$, 20 mM Tris-HCl pH 7.5 at room temperature for 30 min. dNTPs were then added to the solution (1.25 mM). Reaction mix was brought to 50 ^{o}C, then increased by 1°C increments every 1 min to 60 ^{o}C, and held at 60 ^{o}C for another 20 min. cDNA synthesis was terminated by adding 5 M NaOH to a final concentration of 0.25 M followed by incubating at 95 ^{o}C for 3 min. Equal amount of 5 M HCl was used to neutralize the sample, and the resulting cDNAs was purified with Micro Bio-Spin 6 column (Bio-Rad).

Synthesized cDNAs were then PCR amplified by Phusion^{TM} High-Fidelity DNA Polymerase (New England Biolabs) with primers FW-GGAGGTGAGAGTGAGAGTAT and RV-TTCCCTTACCTACCACTTCC. The PCR products were first checked by 2 % agarose gel electrophoresis and then incubated with 4 units of Taq□I restriction enzyme (New England Biolabs) in 1 X CutSmart buffer (New England Biolabs) at 65 ^{o}C for 30 min. The restriction enzyme digestion products were also checked with 2 % agarose gel electrophoresis.

T-A cloning and Sanger sequencing

cDNA synthesized using methods above was PCR amplified by Phusion^{TM} High-Fidelity DNA Polymerase with primers FW-GGAGGTGAGAGTGAGAGTAT and RV-TTCCCTTACCTACCACTTCC. The PCR products was purified by Zymo-Spin column and processed for Sanger sequencing.

For T-A cloning prior to sequencing, TOPOR TA Cloning[R] Kit (Thermo Fisher) was used. The PCR products from cDNA were cloned into TOPOR vector. We randomly picked 30 monoclones for each sample, and plasmids from these clones were prepared for Sanger sequencing with M13 primer.

NgTET1 oxidation, mTET1 oxidation and peroxotungstate reaction of m^{5}C-RNA

NgTET1 was produced according to literature (*Proc. Natl. Acad. Sci. U.S. A.* 112, 4316-4321 (**2015**). For the oxidation of m^{5}C-containing RNA2, 2 μg RNA was incubated in 50 μL solution containing 50 mM MOPS buffer (pH = 6.9), 100 mM ammonium iron (II) sulfate, 1 mM α-ketoglutarate, 2 mM ascorbic acid, 1 mM DTT, 50 mM NaCl, and 5 μM NgTET1 at 37 °C for 1 hour. After that, 4 U of Proteinase K (New England Biolabs) was added to the reaction mixture and incubate at 37 °C for 30 min. The product was purified on Zymo-Spin column.

Mouse TET1 was purchased from . For the oxidation of m^{5}C-containing RNA2, 2 μg RNA was incubated in 50 μL solution containing 50 mM HEPES buffer (pH = 8.0), 100 μM ammonium iron (II) sulfate, 1 mM α-ketoglutarate, 2 mM ascorbic acid, 2.5 mM DTT, 100 mM NaCl, and 15 μg mTET1 at 37 °C for 80 min. After that, 20 μg of Proteinase K (New England Biolabs) was added to the reaction mixture and incubate at 50 °C for 1 hour. The product was purified on Zymo-Spin column.

15 μL Oxidized m^{5}C-containing RNA2 was then treated with 5 μL 1 M aqueous sodium borohydride (Sigma) solution, in order to reduce the over-oxidized f^{5}C to hm^{5}C. After 1 hour of incubation at room temperature in the dark, the reaction was quenched by adding 10 μL 750 mM sodium acetate (Sigma) (pH = 5.2). The product was purified on Zymo-spin column after no further gas was released. The peroxotungstate oxidation of the result RNA was performed according to the method described above. Then, cDNA was synthesized for this sample, and the PCR product was used for restriction enzyme digestion assay or analyzed by Sanger sequencing.

βGT labelling of hm^{5}C in RNA

Hm^{5}C labelling was performed in 20 μl solution containing 50 mM HEPES buffer (pH 8.0), 25 mM $MgCl_2$, 200 μM UDP-Glc (New England Biolabs), and 10 U of βGT (Thermo Fisher), and 2 μg hm^{5}C-containing RNA1 for 2 hours at 37 °C. The product was purified on Zymo-Spin column.

Cell culture and microRNA isolation

293T cells were maintained in high glucose DMEM medium (Gibco) at 37 °C under 5 % CO2 atmosphere. The media was supplemented with 10 % fetal bovine serum, 100 U/mL penicillin, and 100 μg/mL streptomycin (Gibco).

Total microRNA which includes tRNA of the 293T cells was extracted from the cultured cells using PureLink[TM] miRNA Isolation Kit (Invitrogen). After measuring the concentration and checking the quality by agarose gel, the RNA was stored at – 80 °C or proceed to next step.

NgTET1 oxidation, mTET1 oxidation and peroxotungstate reaction of

tRNA from 293T cells

Typically, 2 μg microRNA was used per reaction. To deacylate the tRNA, microRNA sample was first incubated in 50 μL 0.5 M Tris-HCl, pH 9.0 at 37 ^{o}C for 1 hour. The product was purified on Zymo-Spin column. The NgTET1 oxidation, mTET1 oxidation and peroxotungstate reaction of deacylated tRNA were proceed using the method described above. Then, cDNA of the tRNA$^{ASP(GUC)}$ was synthesized using specific stem-loop primer, and the PCR product was analyzed by Sanger sequencing.

Supplementary Tables

Table S1. Oligonucleotides

X = rC or m^5C or hm^5C.

Name	Type	Sequence (5' to 3')	Source
**T7 primer**	DNA	TAATACGACTCACTATAGG	IDT
**T7 template 1**	DNA	GCGGCGTGATGGTCATCATACTTACA TGGCTGTTCGCTATACCTTAATAGAT ATTCTCCCTATAGTGAGTCGTATTA	IDT
**T7 template 2**	DNA	TTCCCTTACCTACCACTTCCATCACGT ACTCATTTCGATATCAATTGTATACAT ACTCTCACTCTCACCTCCCTATAGTGA GTCGTATTA	IDT
**RNA1**	RNA	GGGAGAATATXTATTAAGGTATAGXG AAXAGXXATGTAAGTATGATGAXXA TXAXGXXGX	*In vitro* transcription
**RNA2**	RNA	GGGAGGTGAGAGTGAGAGTATGTAT AXAATTGATATXGAAATGAGTAXGTG ATGGAAGTGGTAGGTAAGGGAA	*In vitro* transcription
**FAM-RT-primer I**	DNA	FAM-TTCCCTTACCTACCACTTCC	IDT
**RT-primer I**	DNA	TTCCCTTACCTACCACTTCC	IDT
**RT-primer II**	DNA	GGAGGTGAGAGTGAGAGTAT	IDT
**Stem-loop primer**	DNA	CTCAACTGGTGTCGTGGAGTCGGCAA TTCAGTTGAGTGGCTCCCCG	IDT
**tRNA RT-primer I**	DNA	CACGTCCTCGTTAGTATAG	IDT
**tRNA RT-primer II**	DNA	TCAACTGGTGTCGTG	IDT

Table S2 Compound-dependent LC-MS/MS parameters used for nucleosides quantification.

RT: retention time, CE: collision energy, CAE: cell accelerator voltage. All the nucleosides were analyzed in the positive mode.

Compound	Precursor Ion (m/z)	Product Ion (m/z)	RT (min)	Delta RT(min)	CE (V)	CAE (V)
rA+H	268	136	14.6	2	10	4
rA+Na	290	158	14.6	2	10	4
rG+H	284	152	9.1	2	10	4
rG+Na	306	174	9.1	2	10	4
rU+H	245	113	3.8	2	10	4
rU+Na	267	135	3.8	2	10	4
rC+H	244	112	2.6	2	10	4
rC+Na	266	134	2.6	2	10	4
m^5C+H	258	126	5.9	2	12	4
m^5C+Na	280	148	5.9	2	12	4
hm^5C+H	274	142	3.1	2	8	4
hm^5C+Na	296	164	3.1	2	8	4

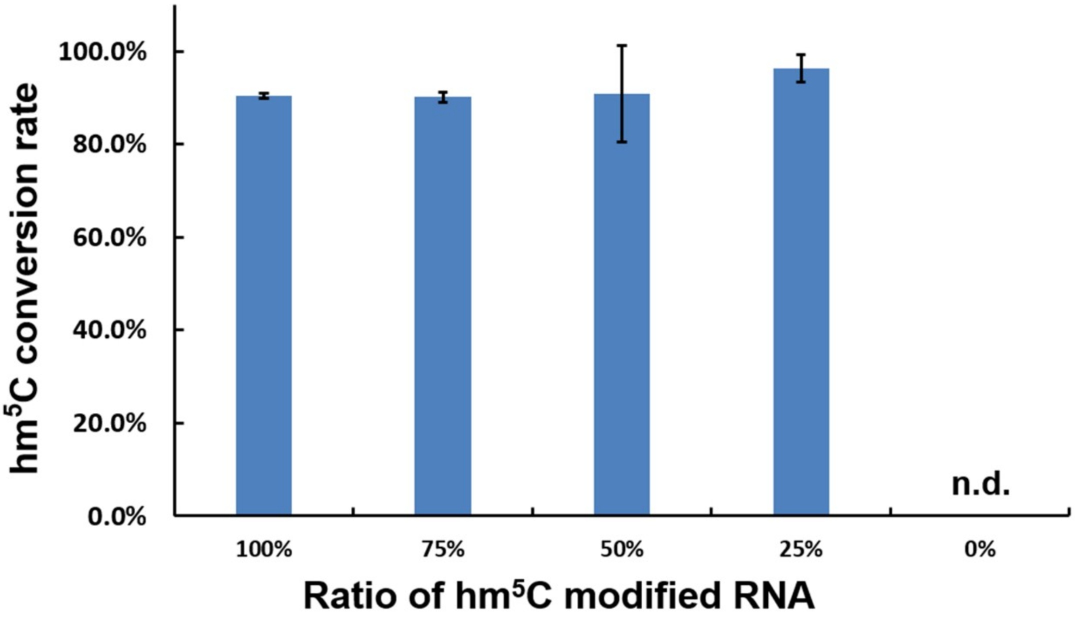

Figure S1. Hm^{5}C conversion rate of different combinations of hm^{5}C modified and unmodified RNA samples.

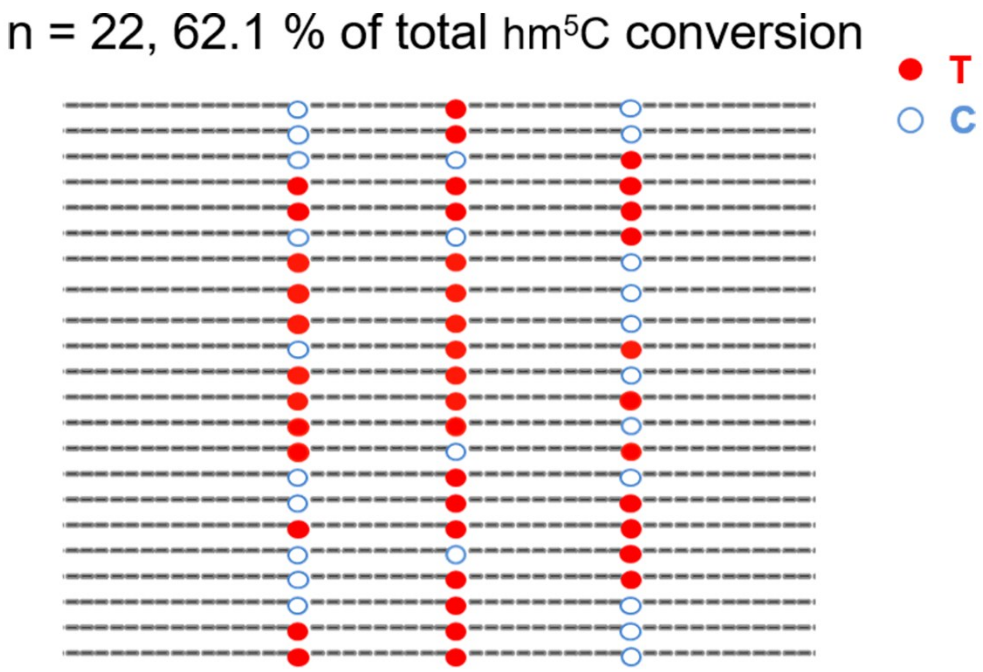

Figure S2. Sanger sequencing results of PCR products from peroxotungstate treated hm^{5}C-containing RNA2. Among 66 hm^{5}C sites sequenced, 41 of them changed to T. The C-to-T conversion rate is 62.1 %.

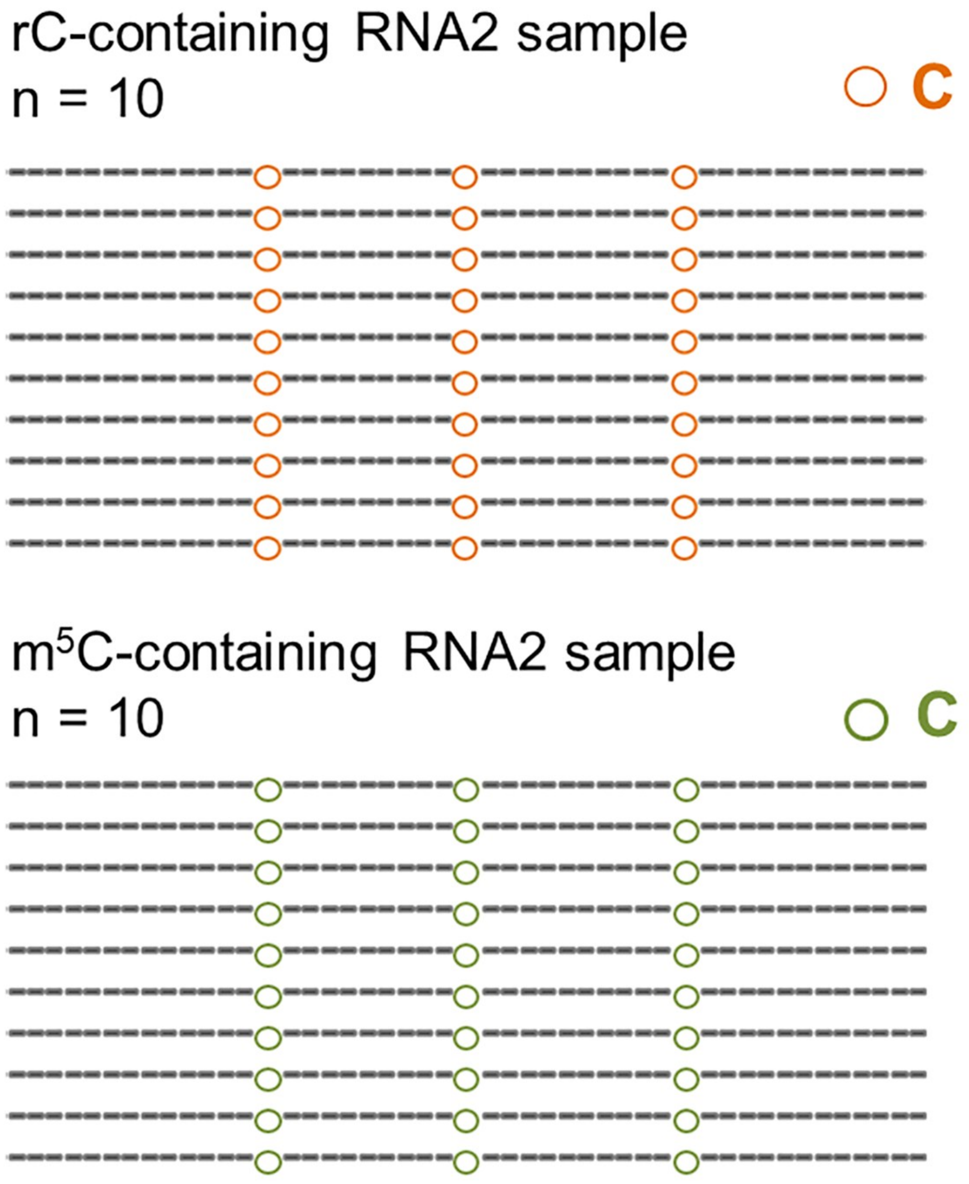

Figure S3. Sanger sequencing results of PCR products from peroxotungstate treated rC-containing RNA2 and m^{5}C-containing RNA2. Among 33 sites sequenced in each sample, no C-to-T transition was detected, indicating the good selectivity of peroxotungstate oxidation on hm^{5}C.

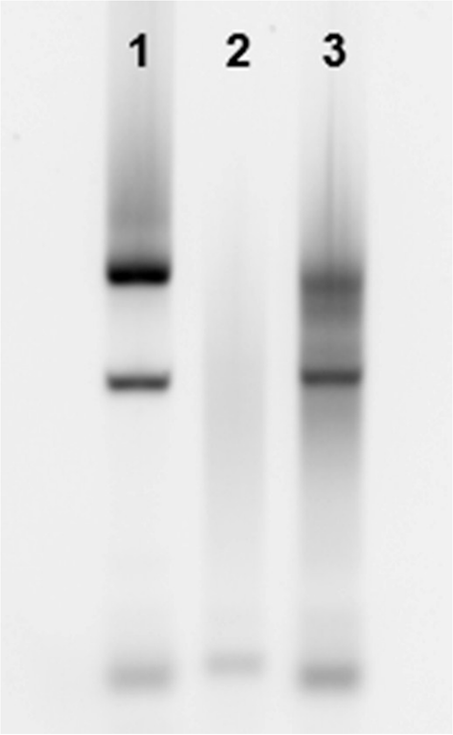

Figure S4. Gel results of (1) control total RNA, (2) total RNA after bisulfite treatment and (3) total RNA after peroxotungstate treatment.

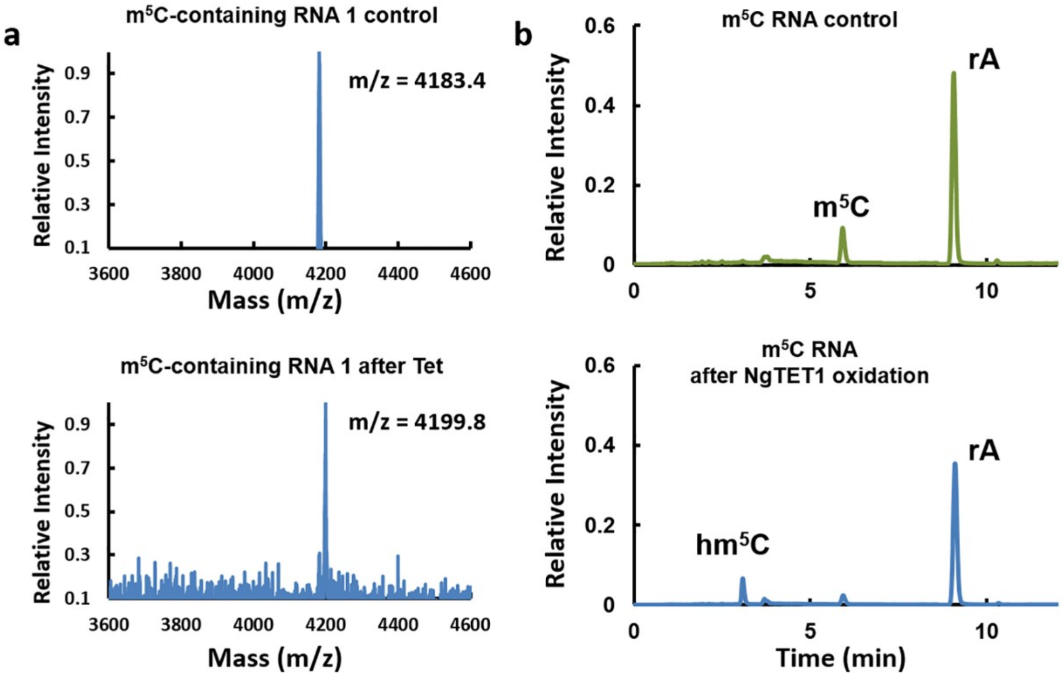

Figure S5. MALDI-MS and HPLC-MS/MS results of m^{5}C-containing RNA1 before and after NgTET1 oxidation. a) In MALDI-MS results, the m/z of original m^{5}C RNA fragment increased 16, indicating the m^{5}C to hm^{5}C change in the RNA. b) After oxidation, a new peak of hm^{5}C appeared in the HPLC-MS/MS.

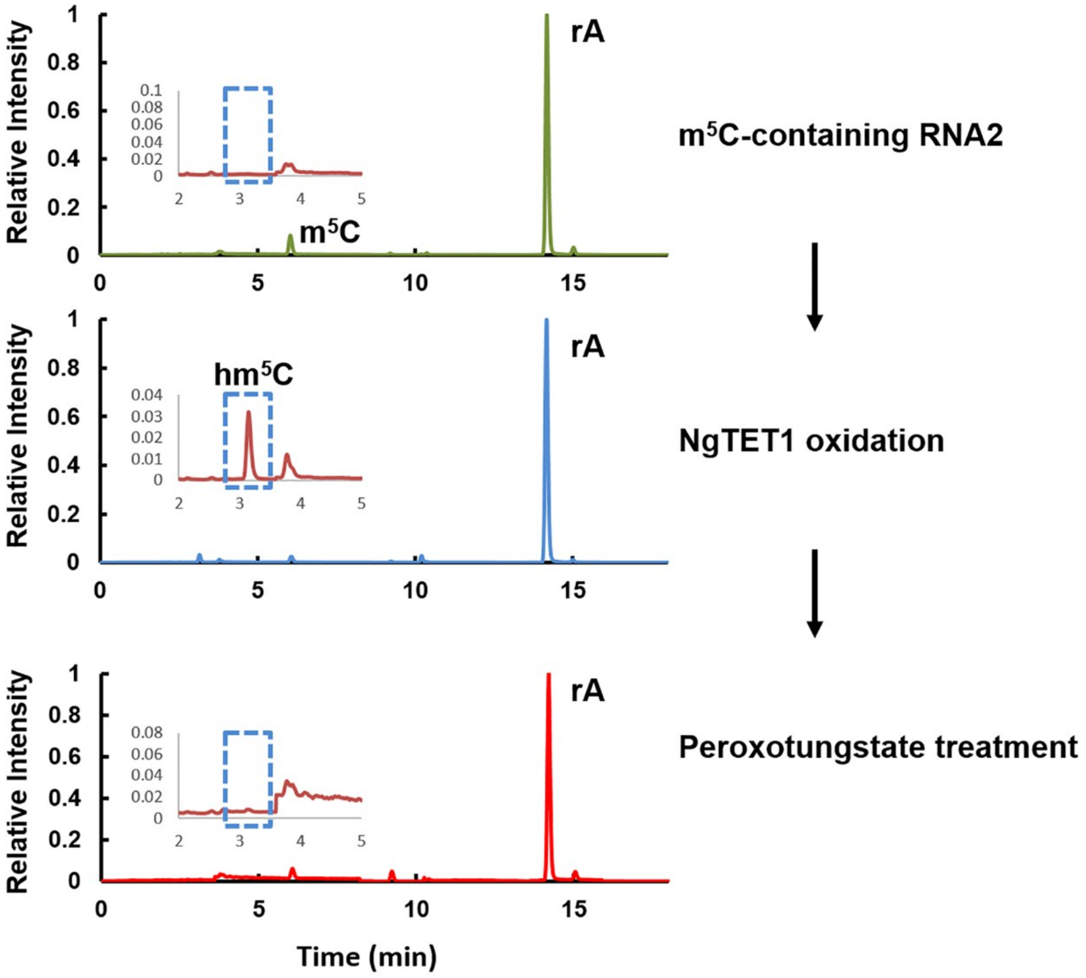

Figure S6. HPLC-MS/MS results of NgTET1-assisted peroxotungstate treated m^{5}C-containing RNA2.

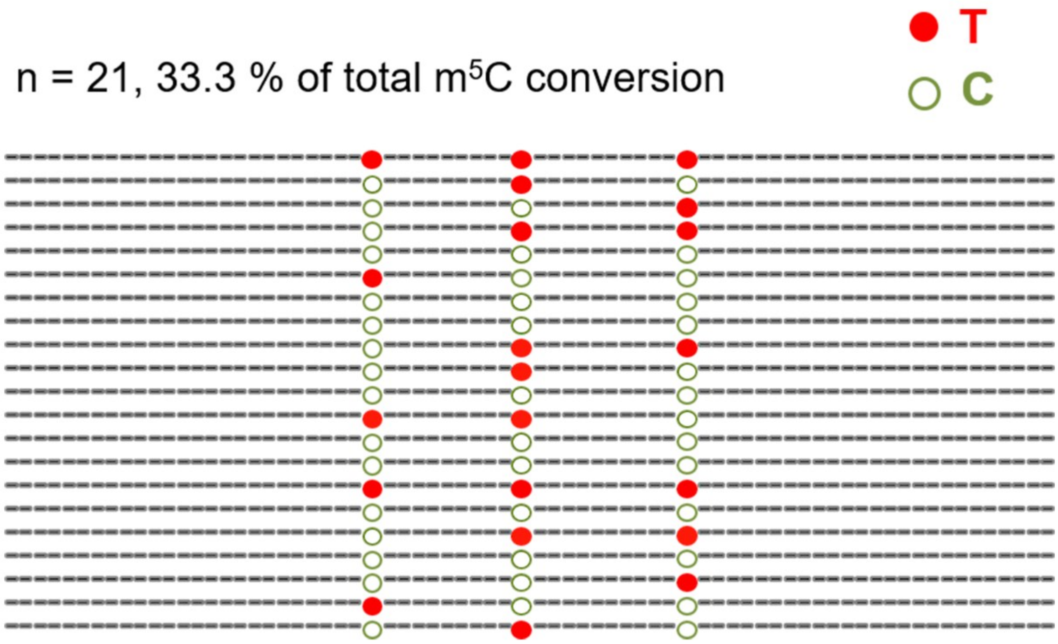

Figure S7. Sanger sequencing analysis of individual PCR products of m^{5}C-containing RNA2 after the NgTET1 assisted peroxotungstate treatment. Among 63 m^{5}C sites sequenced, 21 of them changed to T. The C-to-T conversion rate is 33.3 %.

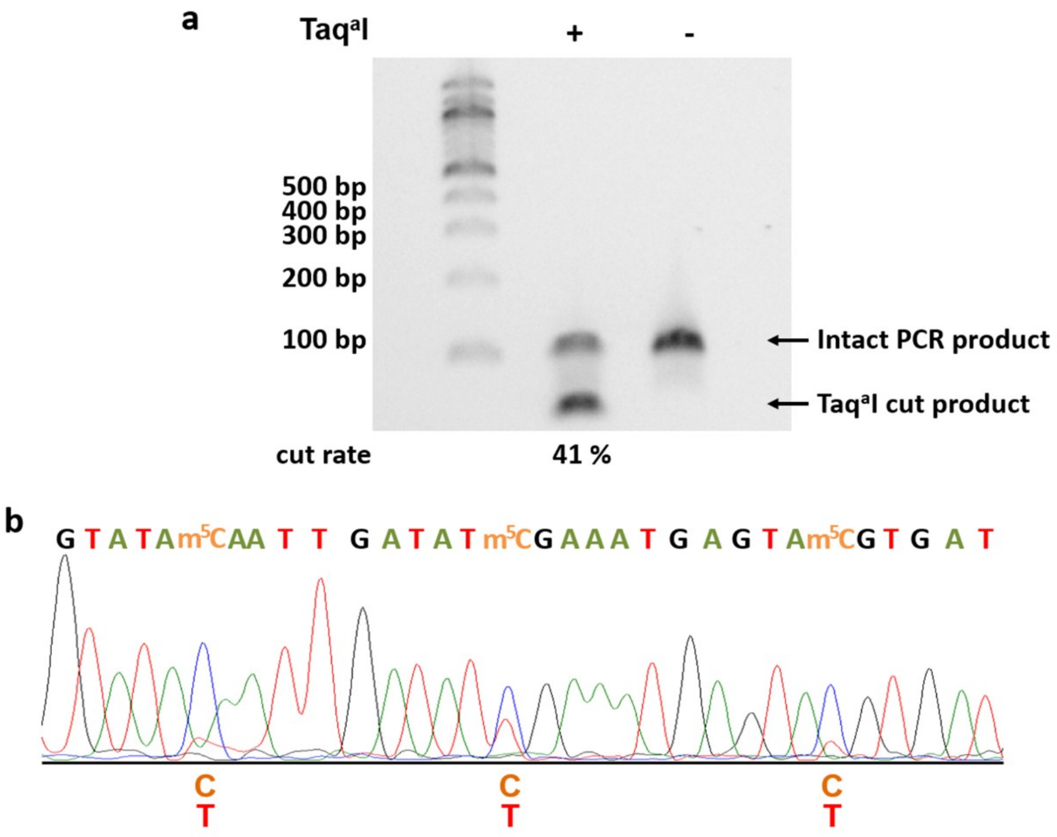

Figure S8. The combination of mTET1 oxidation and peroxotungstate reaction in detecting m^{5}C in model RNA. (a) Restriction enzyme assay result and (b) Sanger sequencing result of m^{5}C-containing RNA2 after the mTET1 assisted peroxotungstate treatment.

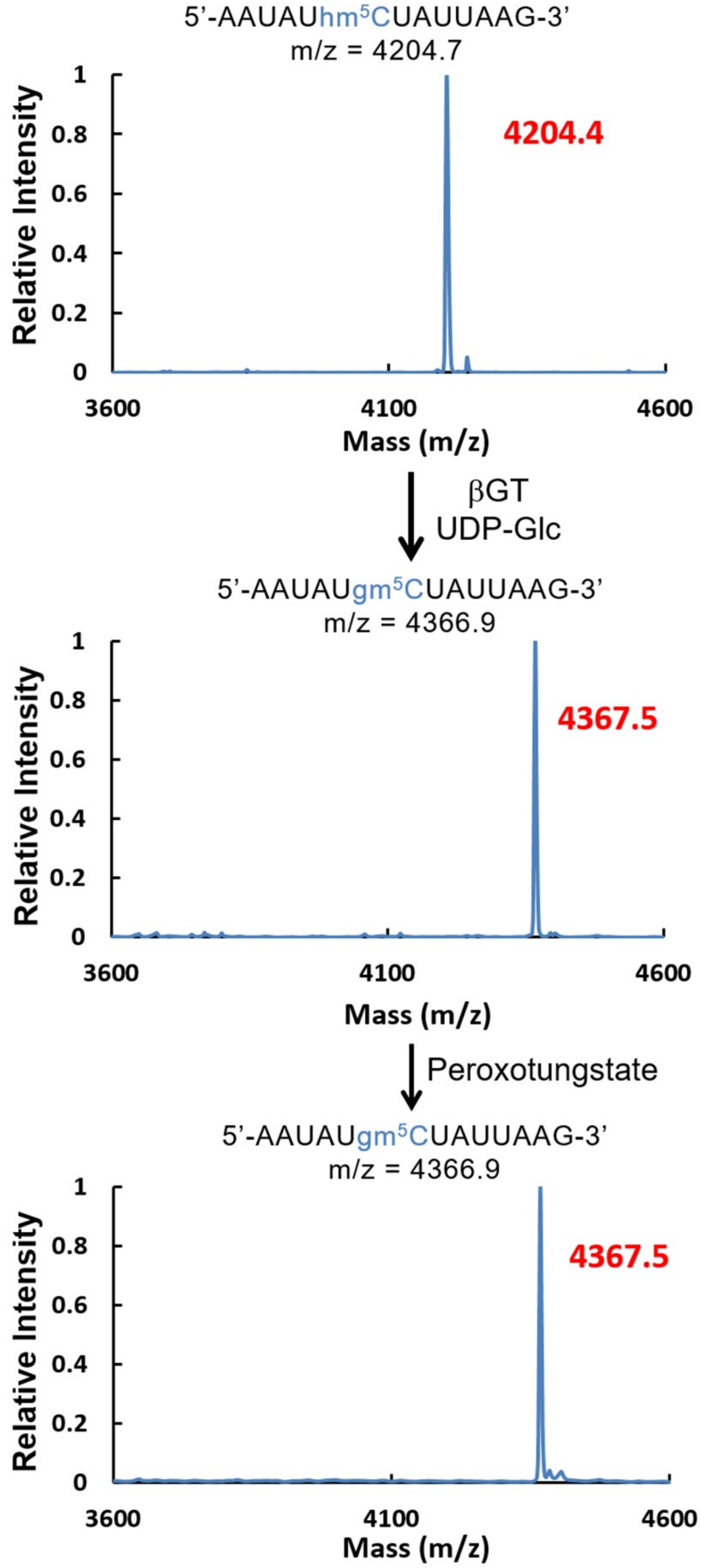

Figure S9. Using β- glucosyltransferase (βGT) to label hm^{5}C with glucose can protect the hm^{5}C-containing RNA from peroxotungstate oxidation. MALDI-MS results of a fragment of hm^{5}C-containing RNA1, RNA1 labelled by glucose with β-glucosytransferase (βGT) and this gm^{5}C RNA after peroxotungstate treatment. Calculated m/z is shown in black, observed m/z is shown in red.

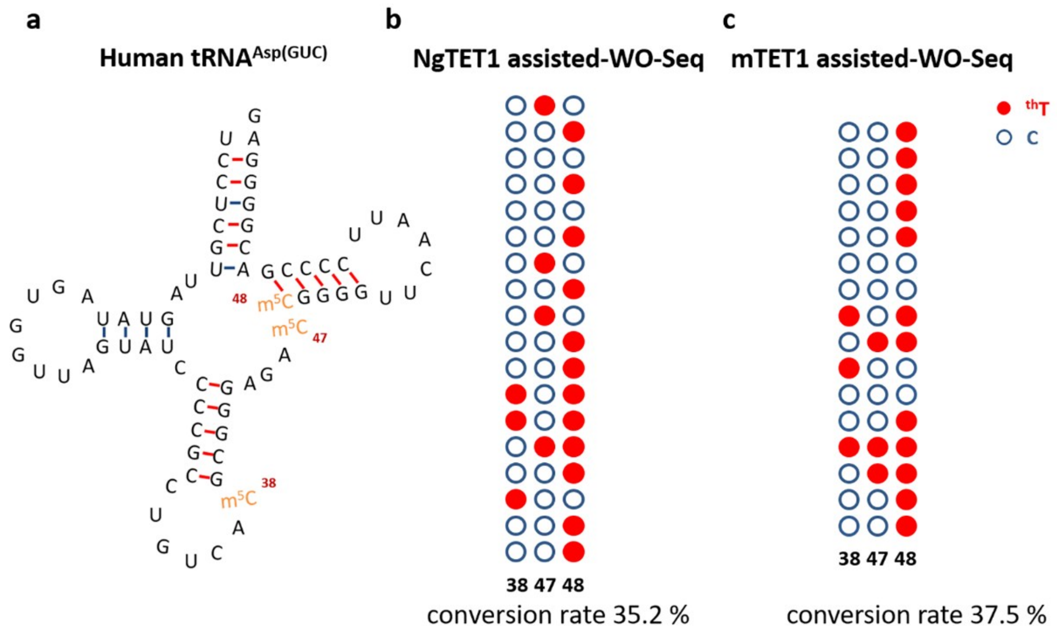

Figure S10. TAWO-Seq results of tRNA$^{Asp(GUC)}$ from 293T cells. (a) Structure of human tRNA$^{Asp(GUC)}$. (b) Sanger sequencing analysis of individual PCR products of tRNA$^{Asp(GUC)}$ after the NgTET1 assisted peroxotungstate treatment. Among 54 m^{5}C sites sequenced, 19 of them changed to T. The C-to-T conversion rate is 35.2 %. (c) Sanger sequencing analysis of individual PCR products of tRNA$^{Asp(GUC)}$ after the mTET1 assisted peroxotungstate treatment. Among 48 m^{5}C sites sequenced, 18 of them changed to T. The C-to-T conversion rate is 37.5 %.
